# Clinical factors associated with the therapeutic efficacy of atezolizumab plus bevacizumab in patients with unresectable hepatocellular carcinoma: A multicenter prospective observational study

**DOI:** 10.1371/journal.pone.0294590

**Published:** 2024-01-02

**Authors:** Machiko Kai, Hayato Hikita, Maesaka Kazuki, Yuki Tahata, Kazuma Shinkai, Akira Doi, Kazuyoshi Ohkawa, Masanori Miyazaki, Hisashi Ishida, Kengo Matsumoto, Yasutoshi Nozaki, Takayuki Yakushijin, Ryotaro Sakamori, Akira Kaneko, Sadaharu Iio, Takatoshi Nawa, Naruyasu Kakita, Naoki Morishita, Naoki Hiramatsu, Takeo Usui, Kazuho Imanaka, Yoshinori Doi, Mitsuru Sakakibara, Yuichi Yoshida, Tsugiko Oze, Takahiro Kodama, Tomohide Tatsumi, Tetsuo Takehara

**Affiliations:** 1 Department of Gastroenterology and Hepatology, Osaka University Graduate School of Medicine, Suita, Osaka, Japan; 2 Department of Hepatobiliary and Pancreatic Oncology, Osaka International Cancer Institute, Osaka, Osaka, Japan; 3 Department of Gastroenterology and Hepatology, Osaka Police Hospital, Osaka, Osaka, Japan; 4 Department of Gastroenterology and Hepatology, Ikeda Municipal Hospital, Ikeda, Osaka, Japan; 5 Department of Gastroenterology and Hepatology, Toyonaka Municipal Hospital, Toyonaka, Osaka, Japan; 6 Department of Gastroenterology and Hepatology, Kansai Rosai Hospital, Amagasaki, Hyogo, Japan; 7 Department of Gastroenterology and Hepatology, Osaka General Medical Center, Osaka, Osaka, Japan; 8 Department of Gastroenterology and Hepatology, National Hospital Organization Osaka National Hospital, Osaka, Osaka, Japan; 9 Department of Gastroenterology and Hepatology, Japan Community Healthcare Organization Osaka Hospital, Osaka, Osaka, Japan; 10 Department of Gastroenterology and Hepatology, Hyogo Prefectural Nishinomiya Hospital, Nishinomiya, Hyogo, Japan; 11 Department of Gastroenterology and Hepatology, Higashiosaka City Medical Center, Higashiosaka, Osaka, Japan; 12 Department of Gastroenterology and Hepatology, Kaizuka City Hospital, Kaizuka, Osaka, Japan; 13 Department of Gastroenterology and Hepatology, Minoh City Hospital, Minoh, Osaka, Japan; 14 Department of Gastroenterology and Hepatology, Osaka Rosai Hospital, Sakai, Osaka, Japan; 15 Department of Gastroenterology and Hepatology, Ashiya Municipal Hospital, Ashiya, Hyogo, Japan; 16 Department of Gastroenterology and Hepatology, Itami City Hospital, Itami, Hyogo, Japan; 17 Department of Gastroenterology and Hepatology, Otemae Hospital, Osaka, Osaka, Japan; 18 Department of Gastroenterology and Hepatology, Yao Municipal Hospital, Yao, Osaka, Japan; 19 Department of Gastroenterology and Hepatology, Suita Municipal Hospital, Suita, Osaka, Japan; 20 Department of Gastroenterology and Hepatology, Koga Community Hospital, Yaidu, Shizuoka, Japan; Kindai University Faculty of Medicine, JAPAN

## Abstract

The treatment efficiency and predictors of atezolizumab plus bevacizumab therapy for unresectable hepatocellular carcinoma in real-world practice have not been established. This study aimed to assess the efficacy and safety of atezolizumab plus bevacizumab and to investigate predictors of progression-free survival and overall survival. Patients with unresectable hepatocellular carcinoma treated with atezolizumab plus bevacizumab therapy in 19 hospitals were enrolled before treatment and observed prospectively. The outcomes of 222 patients in this cohort were analyzed. The objective response rate and disease control rate were 22.0% and 70.6%, respectively, whereas the median progression-free survival was 5.7 months. Independent risk factors for shortened progression-free survival were younger age (<75 years; 3.9 months vs. 8.6 months), higher number of intrahepatic tumors (≥5; 4.0 months vs. 7.9 months), macrovascular invasion (2.3 months vs. 6.7 months), and higher neutrophil-to-lymphocyte ratio (≥3.03; 3.0 months vs. 7.8 months). The median overall survival was not reached; however, independent risk factors for shortened overall survival were absence of hyperlipidemia, higher number of intrahepatic tumors (≥5), macrovascular invasion, higher α-fetoprotein level (≥400 ng/mL), worse Child–Pugh score (≥6), and higher neutrophil-to-lymphocyte ratio (≥3.03). Severe adverse events (grade ≥3) were observed in 96 patients (36.0%), with proteinuria being the most frequent. In conclusion, patients with older age, lower number of intrahepatic tumors, absent macrovascular invasion, and lower neutrophil-to-lymphocyte ratio are expected to have better progression-free survival with atezolizumab plus bevacizumab therapy for unresectable hepatocellular carcinoma.

## Introduction

Hepatocellular carcinoma (HCC) is the most common type of primary liver cancer, and its systemic treatment has rapidly changed. The IMbrave150 trial indicated that the combination of atezolizumab and bevacizumab, an immune-checkpoint inhibitor (ICI) and an anti-vascular endothelial growth factor monoclonal antibody, was superior to sorafenib with respect to overall survival (OS) and progression-free survival (PFS) [1, 2]. Based on the results of the trial, atezolizumab plus bevacizumab therapy has been recommended as the primary systemic treatment for unresectable HCC (uHCC) [3–5].

Recent studies reported the early efficacy and safety of atezolizumab plus bevacizumab in real-world practice and investigated the predictive factors associated with therapeutic outcomes in early experiences [6–9]. Some retrospective studies showed that the CRAFITY score, composed of C-reactive protein and α-fetoprotein (AFP) levels, was useful as an early-time prognostic indicator in patients treated with atezolizumab plus bevacizumab for uHCC [10, 11]. Other studies also demonstrated the utility of neutrophil-to-lymphocyte ratio (NLR) as a clinical index of systemic inflammation associated with early therapeutic response to atezolizumab plus bevacizumab therapy [7, 9]. While these simple clinical indicators are useful, they have only been studied retrospectively in early clinical practice, and clinical indicators associated with the therapeutic efficacy of atezolizumab plus bevacizumab have not been studied prospectively.

This study aimed to assess the efficacy and safety of atezolizumab plus bevacizumab therapy in the real world using a prospective cohort and to investigate the predictors of PFS and OS in patients receiving atezolizumab plus bevacizumab therapy for uHCC.

## Materials and methods

### Patients

**Fig 1:**
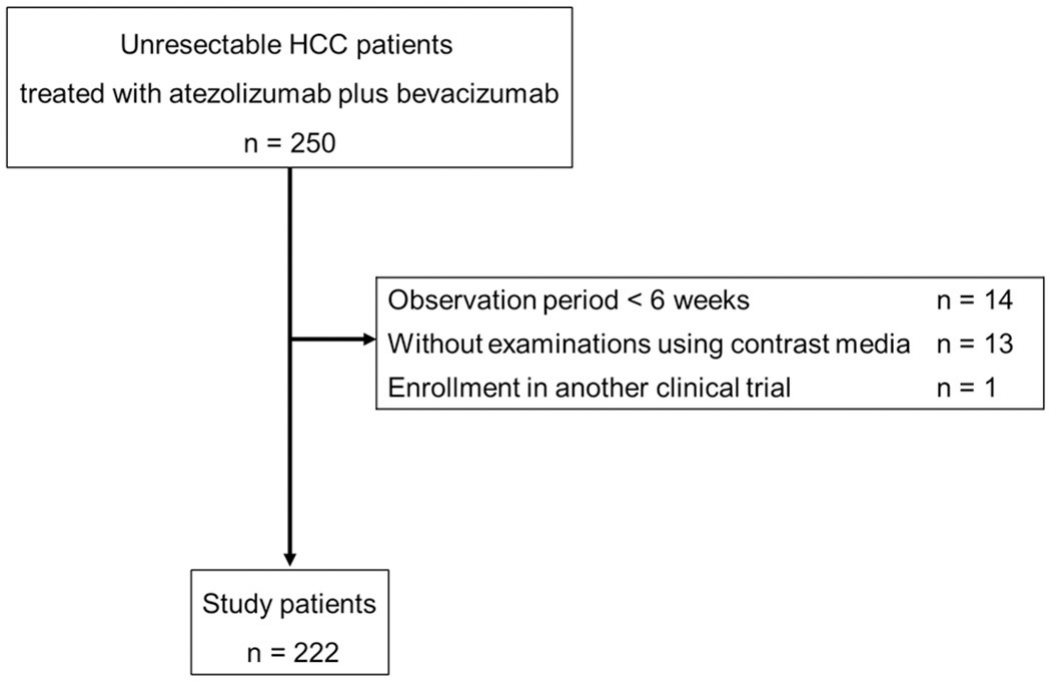
Flowchart of study enrollment.

In total, 250 patients with uHCC treated with atezolizumab plus bevacizumab from November 2020 to August 2022 at Osaka University Hospital and 18 affiliated hospitals in the Osaka Liver Forum were enrolled before commencing treatment and were prospectively observed. At hospitals in the Osaka Liver Forum, patients with uHCC deemed unsuitable for surgical resection, liver transplantation, radiofrequency ablation, or transarterial chemoembolization were considered to be eligible for atezolizumab plus bevacizumab therapy. In some cases, patients with Barcelona Clinic Liver Cancer stage A were also considered to be eligible for treatment. In principle, patients with Child–Pugh class A were treated; nevertheless, depending on each attending physicians’ judgement, some patients with Child–Pugh class B were also treated. The eligibility criteria for this prospective cohort were as follows: (1) patients treated with atezolizumab plus bevacizumab therapy at hospitals in the Osaka Liver Forum; (2) patients with Eastern Cooperative Oncology Group performance status of 0 or 1; and (3) patients who consented to this study. The exclusion criteria were as follows: (1) patients with an observation period of <6 weeks; (2) patients who did not undergo examinations using contrast media; and (3) patients who were enrolled in another clinical trial (Fig 1). Among the 250 patients, 222 patients were included in the analysis.

The Ethics Committee of Osaka University Hospital (UMIN-000034611) and each participating institution approved the study protocol. Written informed consent was obtained from all participants at each institute.

### Treatment with atezolizumab plus bevacizumab and assessment of adverse events (AEs)

All 222 patients intravenously received atezolizumab 1200 mg plus bevacizumab 15 mg/kg once every 3 weeks. If any unacceptable or serious AE related to the drug occurred, the administration was interrupted until symptoms diminished to grade 1 or 2. The patients continued treatment until the treatment failed or an unacceptable AE occurred. The relative intensity of bevacizumab was defined as the ratio of the amount of the actual dose to the standard dose until the treatment was discontinued. Data on AEs were collected during treatment, and AEs were evaluated according to the National Cancer Institute Common Terminology Criteria for Adverse Events version 4.0.

### Assessment of hepatic function

The prothrombin time in patients who took oral warfarin was assigned a score of 1 point to calculate the Child–Pugh score [12, 13]. The albumin-bilirubin (ALBI) score was calculated using the following formula; (log_10_ (total bilirubin [mg/dL] ×17.1) ×0.66) + (albumin [g/dL] × 10 × -0.085) [14]. In addition, the modified ALBI (mALBI) grade was used to categorize liver function [15].

### Assessment of therapeutic efficacy

Imaging studies, such as contrast-enhanced computed tomography and magnetic resonance imaging, were performed every 6–8 weeks. The therapeutic response was assessed on the Response Evaluation Criteria in Solid Tumor version 1.1 (RECIST version 1.1) [16] and modified RECIST (mRECIST) in each institution [17]. The objective response rate (ORR) was defined as the sum of the percentage of complete response and partial response. The disease control rate (DCR) was defined as the sum of the percentage of complete response, partial response, and stable disease. PFS was the time from the start date of treatment to the date of progressive disease or death. OS the time from the start date of treatment to the date of death or patients’ last follow-up date.

### Statistical analysis

Patient characteristics were expressed as medians and interquartile ranges (IQRs) for continuous variables and as actual numbers and percentages for categorical variables. OS and PFS were calculated using the Kaplan–Meier method, and their statistical differences were evaluated using the log-rank test. Cox proportional hazard models were used to identify independent factors associated with PFS and OS.

The NLR was calculated by dividing the absolute neutrophil count by the absolute lymphocyte count from peripheral complete blood counts. The cutoff value of NLR’s predictive capability for radiological progressive disease was calculated using a receiver operating characteristic curve based on sensitivity and specificity using the Youden index [18]. Cutoff values for other factors such as age [19], AFP level [1], maximum tumor diameter, number of tumors [19, 20], Child–Pugh score, and mALBI grade [7, 21, 22] were based on previous studies.

Statistical significance was set at a P value of < 0.05. All analyses were performed using SPSS statistical software version 24.0 for Windows (IBM Corp., Armonk, NY, USA).

## Results

### Patient characteristics

Table 1 shows the baseline characteristics of the study participants, as well as the characteristics according to the treatment line. The median age of patients was 73 years, and 176 (79.3%) study patients were men. One hundred and ten (49.5%), 96 (43.2%), 15 (6.8%), and 1 (0.5%) patient had Child–Pugh scores of 5, 6, 7, and 8, respectively. One hundred and six (47.7%) patients had more than four intrahepatic tumors, 37 (16.7%) patients had macrovascular invasion, and 88 (39.6%) patients had extrahepatic metastasis. Among all patients, 23 had no data on gastroesophageal varices prior to treatment; 111 (50.0%), 64 (28.8%), and 24 (10.8%) patients had F0, F1, and F2 gastroesophageal varices, respectively. The median NLR was 2.44 (IQR: 1.78–3.55). One hundred and forty-seven (66.2%) patients received atezolizumab plus bevacizumab as their first systemic chemotherapy, whereas 75 (33.8%) received another regimen before treatment. Table 1 also shows the characteristics according to the treatment line. The median observation period was 9.9 months.

**Table 1:** Patient characteristics.

Characteristic		All	First-line systemic treatment	Second- or later-line systemic treatment
		n = 222		
			n = 147	n = 75
Age, years	Median (IQR)	73 (66–79)	75.0 (67–80)	71 (62–76)
Sex, n (%)	Male	176 (79.3)	116 (78.9)	60 (80.0)
	Female	46 (20.7)	31 (21.1)	15 (20.0)
Hypertension, n (%)	Absent	67 (30.2)	47 (32.0)	20 (26.7)
	Present	155 (69.8)	100 (68.0)	55 (73.3)
Diabetes mellitus, n (%)	Absent	135 (60.8)	82 (55.8)	53 (70.7)
	Present	87 (39.2)	65 (44.2)	22 (29.3)
Hyperlipidemia, n (%)	Absent	182 (82.0)	120 (81.6)	62 (82.7)
	Present	40 (18.0)	27 (18.4)	13 (17.3)
ECOG PS, n (%)	0	206 (92.8)	138 (93.9)	68 (90.7)
	1	16 (7.2)	9 (6.1)	7 (9.3)
Prior systemic therapy, n (%)	Absent	147 (66.2)	-	-
	Present	75 (33.8)		
Etiology, n (%)	Viral	120 (54.1)	68 (46.3)	52 (69.3)
	Non-viral	102 (45.9)	79 (53.7)	23 (30.7)
Child–Pugh score classification, n (%)	5	110 (49.5)	74 (50.3)	36 (48.0)
	6 or 7 or 8	112 (50.5)	73 (49.7)	39 (52.0)
ALBI score	Median (IQR)	-2.35 (-2.65 to -2.01)	-2.34 (-2.61 to -2.06)	-2.35 (-2.70 to -2.02)
mALBI grade, n (%)	1 or 2a	127 (57.2)	81 (55.1)	46 (48.0)
	2b or 3	95 (42.8)	66 (44.9)	29 (39.7)
Maximum intrahepatic tumor size, mm	Median (IQR)	25.5 (16.0–46.3)	26.0 (16.0–51.0)	25.0 (14.0–37.0)
Intrahepatic tumor number, n (%)	≤4	116 (52.3)	79 (53.7)	37 (49.3)
	≥ 5	106 (47.7)	68 (46.3)	38 (50.7)
Macrovascular invasion, n (%)	Absent	185 (83.3)	122 (83.0)	63 (84.0)
	Present	37 (16.7)	25 (17.0)	12 (16.0)
Extrahepatic metastasis, n (%)	Absent	134 (60.4)	92 (62.6)	42 (56.0)
	Present	88 (39.6)	55 (37.4)	33 (44.0)
Gastroesophageal varices, n (%)	F0	111 (50.0)	71 (48.3)	40 (53.3)
	F1	64 (28.8)	39 (26.5)	25 (33.3)
	F2	24 (10.8)	17 (11.6)	7 (9.3)
	no data	23 (10.4)	20 (13.6)	3 (4.0)
Barcelona Clinic Liver Cancer stage, n (%)	A or B	109 (49.1)	73 (49.7)	36 (48.0)
	C	113 (50.9)	74 (50.3)	39 (52.0)
AFP, ng/mL	Median (IQR)	17 (4–747)	17 (4–398)	20 (4–1032)
NLR	Median (IQR)	2.44 (1.78–3.55)	2.55 (1.78–3.73)	2.29 (1.78–3.37)

ECOG PS, Eastern Cooperative Oncology Group performance status; ALBI, albumin-bilirubin; mALBI, modified albumin-bilirubin; AFP, α-fetoprotein; NLR, neutrophil-to-lymphocyte ratio.

### Efficacy of atezolizumab plus bevacizumab therapy

**Fig 2:**
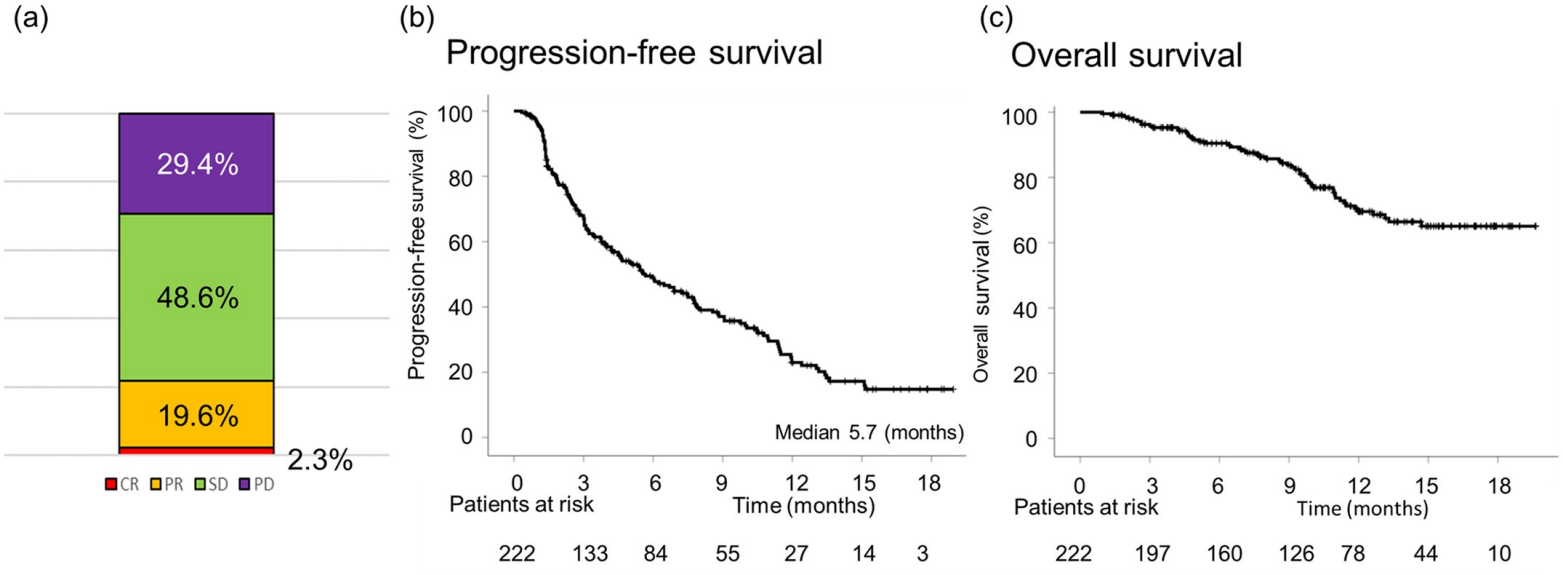
(a) Therapeutic efficacy of atezolizumab plus bevacizumab therapy. (b) PFS and (c) OS of all patients receiving atezolizumab plus bevacizumab therapy.

Fig 2a presents the best therapeutic responses based on RECIST version 1.1. The ORR and DCR were 22.0% and 70.6%, respectively. Eight patients were not evaluated after the treatment commenced. During the observation period, 133 patients exhibited disease progression, and 53 patients in the whole cohort died. Among these 53 patients, death was due to liver-related diseases in 48 patients, infection in two patients, and HCC rupture in one patient, immune-related AE (hepatotoxicity) in one patient, and gastrointestinal perforation in one patient. The median PFS was 5.7 months, and the median OS was not yet reached (Fig 2b and 2c). Focusing on the first-line systemic treatment group, the best therapeutic responses based on RECIST version 1.1 were similar (S1a Fig). The median PFS was 7.8 months, and the median OS had not yet reached (S1b and S1c Fig). The therapeutic efficacy in all patients and the first-line treatment group based on mRECIST was compatible with those based on RECIST version 1.1 (S2 Fig).

In the entire cohort, 151 patients discontinued atezolizumab plus bevacizumab therapy because of progressive disease and AEs. Out of these 151 patients, 61 (40.4%), 29 (19.2%), 15 (9.9%), and 46 (30.5%) received lenvatinib, other molecular target agents, other treatments such as transarterial chemoembolization and radiation therapy, and best supportive care, respectively.

### Clinical factors associated with PFS

**Fig 3:**
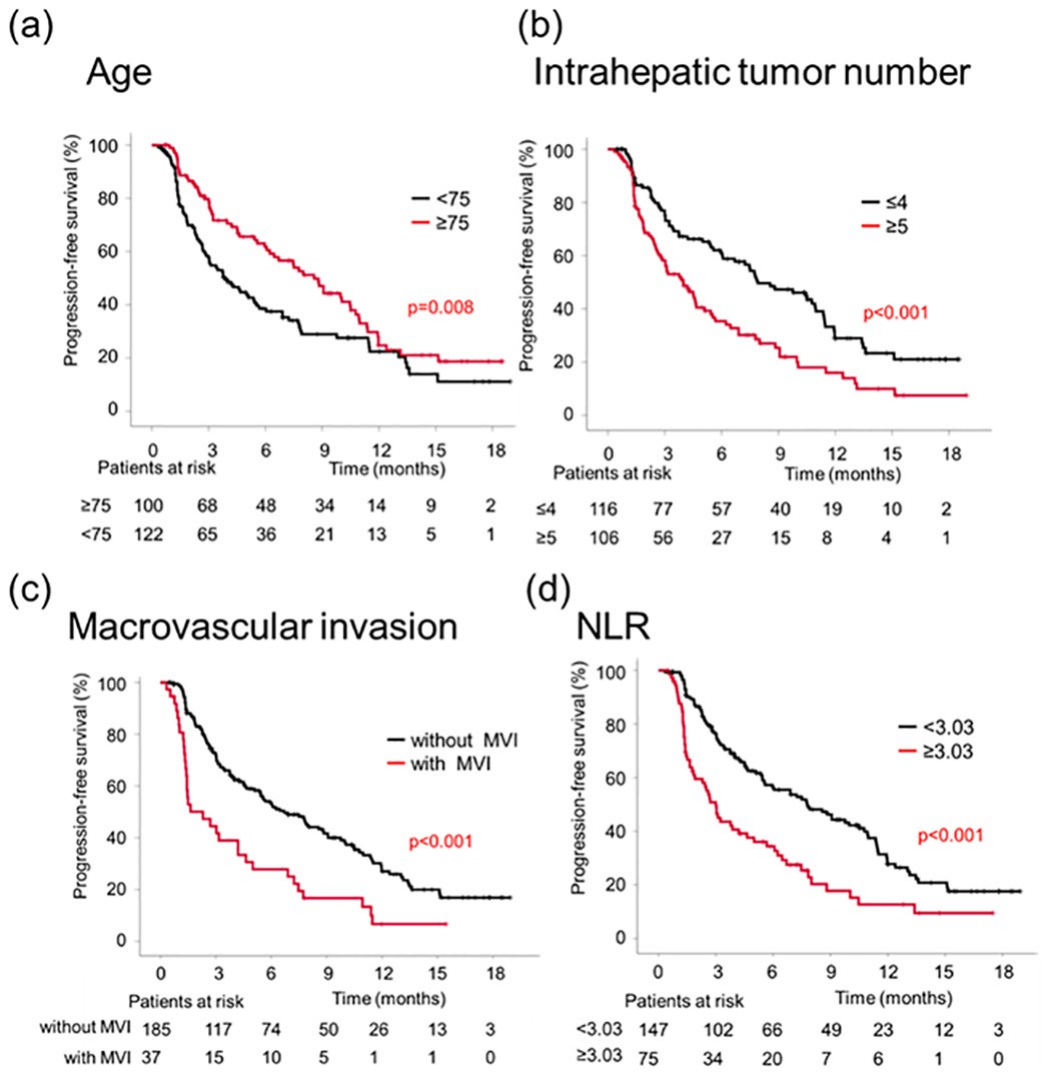
Kaplan–Meier curves for PFS according to age (a), intrahepatic tumor number (b), macrovascular invasion (c), and NLR (d).

Pretreatment clinical information was analyzed to investigate risk factors for the shortened PFS based on RECIST version 1.1. Univariate analysis identified age, maximum intrahepatic tumor size, number of intrahepatic tumors, macrovascular invasion, and NLR as significant factors associated with PFS. In contrast, hepatitis etiology or prior systemic therapy was not associated with PFS. Multivariate analysis identified younger age, a higher number of intrahepatic tumors, macrovascular invasion, and higher NLR as independent factors associated with the shorter PFS (Table 2). The median PFS was shorter in the group with younger age (<75 years; 3.9 months vs. 8.6 months), higher number of intrahepatic tumors (≥5; 4.0 months vs. 7.9 months), macrovascular invasion (2.3 months vs. 6.7 months), and higher NLR (≥3.03; 3.0 months vs. 7.8 months) than in the other groups (Fig 3).

**Table 2:** Univariate and multivariate analyses of PFS-related factors.

Variable	Category	Univariate analysis	P-value	Multivariate analysis	P-value
		Hazard ratio (95% CI)		Hazard ratio (95% CI)	
Age, years	<75	1		1	
	≥75	0.645 (0.464–0.895)	0.009	0.674 (0.484–0.939)	0.02
Sex	Male	1			
	Female	1.036 (0.700–1.533)	0.860		
Hypertension	Absent	1			
	Present	0.920 (0.652–1.297)	0.632		
Diabetes mellitus	Absent	1			
	Present	0.946 (0.677–1.321)	0.745		
Hyperlipidemia	Absent	1			
	Present	1.023 (0.673–1.555)	0.914		
Prior systemic therapy	Absent	1			
	Present	1.360 (0.974–1.901)	0.071		
Etiology	Viral	1			
	Non-viral	1.006 (0.902–1.121)	0.917		
ECOG PS	0	1			
	1	0.981 (0.530–1.814)	0.951		
Maximum intrahepatic tumor size, mm	<50	1		1	
	≥50	1.546 (1.076–2.222)	0.018	1.023 (0.693–1.509)	0.91
Intrahepatic tumor number	≤4	1		1	
	≥5	1.832 (1.320–2.542)	<0.001	1.879 (1.339–2.637)	<0.001
Macrovascular invasion	Absent	1		1	
	Present	2.226 (1.509–3.285)	<0.001	2.266 (1.511–3.397)	<0.001
Extrahepatic metastasis	Absent	1			
	Present	1.115 (0.803–1.549)	0.516		
AFP, ng/mL	<400	1			
	≥400	1.171 (0.821–1.671)	0.383		
Child–Pugh score	5	1			
	6 or 7	1.045 (0.757–1.443)	0.789		
mALBI grade	1 or 2a	1			
	2b or 3	1.146 (0.828–1.586)	0.412		
NLR	<3.03	1		1	
	≥3.03	2.038 (1.457–2.851)	<0.001	1.812 (1.273–2.577)	0.001

CI, confidence interval; PFS, progression-free survival; ECOG PS, Eastern Cooperative Oncology Group performance status; ALBI, albumin-bilirubin; mALBI, modified albumin-bilirubin; AFP, α-fetoprotein; NLR, neutrophil-to-lymphocyte ratio.

### Clinical factors associated with OS

**Fig 4:**
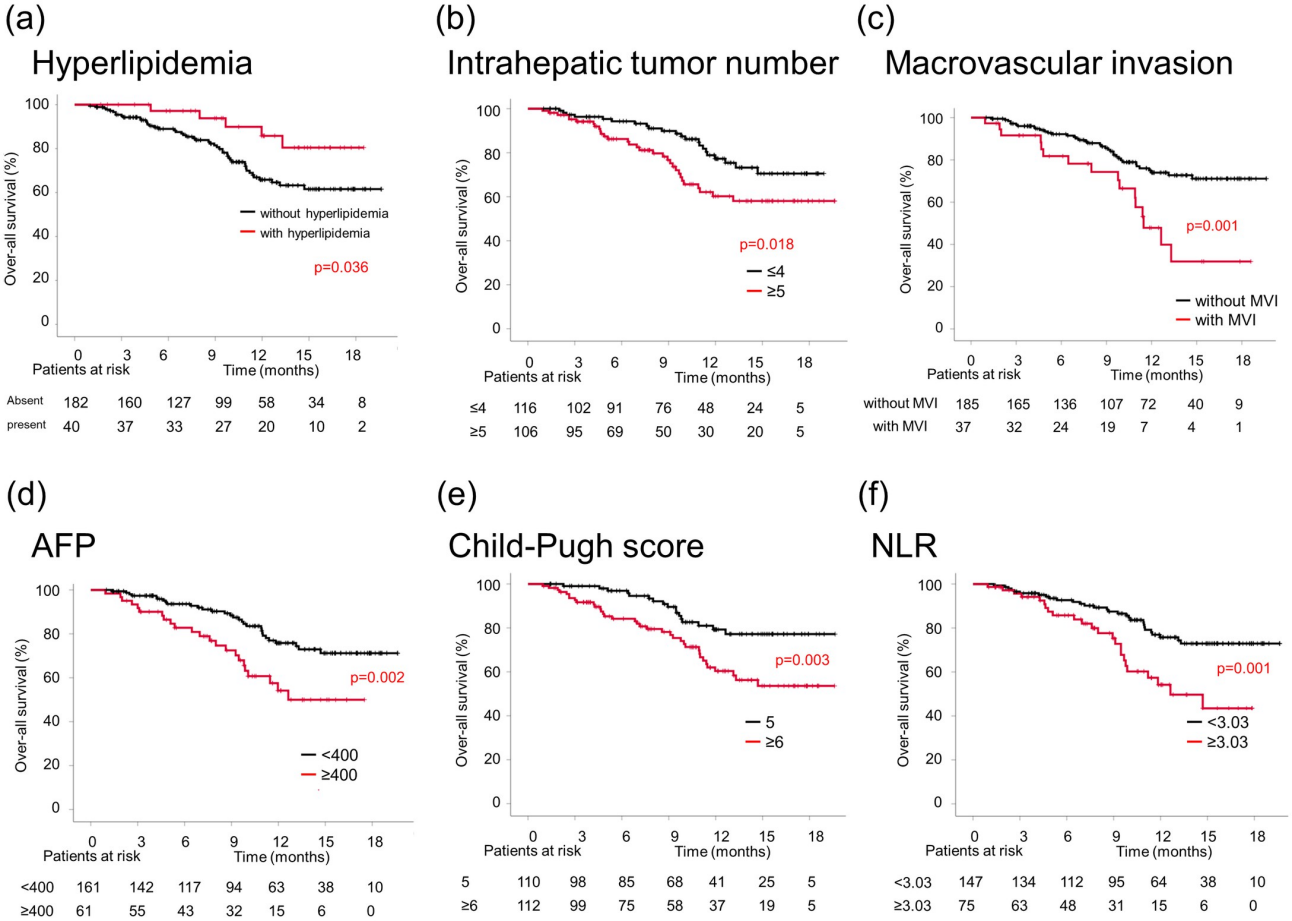
Kaplan–Meier curves for OS according to hyperlipidemia (a), intrahepatic tumor number (b), macrovascular invasion (c), AFP level (d), Child–Pugh score (e), and NLR (f).

Univariate analysis for OS showed that hyperlipidemia, maximum intrahepatic tumor size, several intrahepatic tumors, macrovascular invasion, AFP level, Child–Pugh score, mALBI grade, and NLR were significant factors associated with OS (Table 3). The Child–Pugh score and mALBI grade were related; however, only the Child–Pugh score was included in the multivariate analysis. Multivariate analysis revealed that the absence of hyperlipidemia, higher number of intrahepatic tumors, macrovascular invasion, higher AFP level, worse Child–Pugh score, and higher NLR were significantly associated with poor prognoses (Table 3). The median OS was shorter in the group without hyperlipidemia (not reached vs. not reached), higher number of intrahepatic tumors (≥5; not reached vs. not reached), macrovascular invasion (11.5 months vs. not reached), higher AFP level (≥400 ng/mL; 12.6 months vs. not reached), worse Child–Pugh score (≥6; not reached vs. not reached), and higher NLR (≥3.03; 12.6 months vs. not reached) than in the other groups (Fig 4).

**Table 3:** Univariate and multivariate analyses of OS-related factors.

Variable	Category	Univariate analysis	P-value	Multivariate analysis	P-value
		Hazard ratio (95% CI)		Hazard ratio (95% CI)	
Age, years	<75	1			
	≥75	1.077 (0.628–1.847)	0.786		
Sex	Male	1			
	Female	1.444 (0.784–2.661)	0.239		
Hypertension	Absent	1			
	Present	0.696 (0.397–1.220)	0.206		
Diabetes mellitus	Absent	1			
	Present	0.810 (0.458–1.430)	0.467		
Hyperlipidemia	Absent	1		1	
	Present	2.581 (1.027–6.487)	0.044	2.700 (1.055–6.911)	0.038
Prior systemic therapy	Absent	1			
	Present	1.100 (0636–1.902)	0.733		
Etiology	Viral	1			
	Non-viral	0.948 (0.789–1.139)	0.568		
ECOG PS	0	1			
	1	2.097 (0.946–4.647)	0.068		
Maximum intrahepatic tumor size, mm	<50	1		1	
	≥50	2.438 (1.387–4.285)	0.002	1.479 (0.791–2.764)	0.220
Intrahepatic tumor number	≤4	1		1	
	≥5	1.950 (1.110–3.321)	0.02	2.084 (1.161–3.740)	0.014
Macrovascular invasion	Absent	1		1	
	Present	2.569 (1.426–4.628)	0.002	2.128 (1.146–3.951)	0.017
Extrahepatic metastasis	Absent	1			
	Present	1.122 (0.652–1.932)	0.678		
AFP, ng/mL	<400	1		1	
	≥400	2.307 (1.337–3.982)	0.003	2.422 (1.371–4.280)	0.002
Child–Pugh score	5	1		1	
	6 or 7	2.370 (1.331–4.221)	0.003	2.942 (1.594–5.429)	0.001
mALBI grade	1 or 2a	1			
	2b or 3	1.982 (1.15–13.413)	0.014		
NLR	<3.03	1		1	
	≥3.03	2.361 (1.368–4.037)	0.002	1.920 (1.072–3.437)	0.028

CI, confidence interval; OS, overall survival; ECOG PS, Eastern Cooperative Oncology Group performance status; ALBI, albumin-bilirubin; mALBI, modified albumin-bilirubin; AFP, α-fetoprotein; NLR, neutrophil-to-lymphocyte ratio.

### Treatment-related AEs

Overall, 209 (94.1%) patients experienced AEs, with 80 (36.0%) experiencing severe AEs (grade ≥3). No significant differences in the frequency of serious AEs were observed, irrespective of the presence or absence of each comorbidity and prior systemic therapy (S1 Table). The most frequent AE was proteinuria (43.2%), followed by hypertension (31.1%), fatigue (30.2%), decreased appetite (22.1%), fever (20.7%), bleeding (20.3%), rash (18.5%), increased aspartate aminotransferase or alanine aminotransferase levels (15.8%), thyroid dysfunction (14.4%), and diarrhea (9.9%). The most frequent AE of grade ≥3 was proteinuria (12.6%) (Table 4). In total, 23 (10.4%) patients received systemic steroid treatment for immune-related AEs. The indications for systemic steroid use included interstitial pneumonia (n = 7), liver injury (n = 5), skin lesions (n = 4), colitis (n = 1), pancreatitis (n = 1), pleurisy (n = 1), persistent fever of unknown origin (n = 1), rhabdomyolysis (n = 1), Guillen-Barre syndrome (n = 1), and disseminated intravascular coagulation (n = 1). The frequency of AEs in the first-line systemic treatment group was similar to that in the later-line systemic treatment group (Table 4).

**Table 4:** Treatment-related adverse events.

Treatment-related adverse events	All n = 222	First-line systemic treatment n = 147	Second- or later-line systemic treatment n = 75
Any adverse event			
Any grade, n (%)	209 (94.1)	139 (94.6)	70 (93.3)
Grade ≥3, n (%)	80 (36.0)	46 (31.3)	34 (43.0)
Proteinuria			
Any grade, n (%)	96 (43.2)	60 (40.8)	36 (48.0)
Grade ≥3, n (%)	28 (12.6)	10 (6.8)	18 (22.8)
Hypertension			
Any grade, n (%)	69 (31.1)	54 (36.7)	15 (20.0)
Grade ≥3, n (%)	13 (5.9)	11 (7.5)	2 (2.5)
Fatigue			
Any grade, n (%)	67 (30.2)	46 (31.3)	21 (28.0)
Grade ≥3, n (%)	6 (2.7)	5 (3.4)	1 (1.3)
Decreased appetite			
Any grade, n (%)	49 (22.1)	37 (25.2)	12 (16.0)
Grade ≥3, n (%)	7 (3.2)	6 (4.1)	1(1.3)
Fever			
Any grade, n (%)	46 (20.7)	26 (17.7)	20 (26.7)
Grade ≥3, n (%)	1 (0.5)	0 (0)	1 (1.3)
Bleeding			
Any grade, n (%)	45 (20.3)	29 (19.7)	16 (21.3)
Grade ≥3, n (%)	15 (6.8)	7 (4.8)	8 (10.1)
Rash			
Any grade, n (%)	41 (18.5)	26 (17.7)	15 (20.0)
Grade ≥3, n (%)	3 (1.4)	3 (2.0)	0 (0)
Increased AST or ALT			
Any grade, n (%)	35 (15.8)	22 (15.0)	13 (17.3)
Grade ≥3, n (%)	8 (3.6)	5 (3.4)	3 (3.8)
Hypothyroidism			
Any grade, n (%)	32 (14.4)	19 (12.9)	13 (17.3)
Grade ≥3, n (%)	0 (0)	0 (0)	0 (0)
Diarrhea			
Any grade, n (%)	22 (9.9)	14 (9.5)	8 (10.7)
Grade ≥3, n (%)	3 (0)	1 (0.7)	2 (2.5)
Hoarse voice			
Any grade, n (%)	19 (8.6)	16 (10.9)	3 (4.0)
Grade ≥3, n (%)	0 (0)	0 (0)	0 (0)

AST, aspartate aminotransferase; ALT, alanine aminotransferase.

In the entire cohort, the median relative intensity of bevacizumab during the initial 6 months was 100% (IQR: 87.5–100%). The median relative intensity did not differ between patients with and without hypertension (100% vs. 100%, P = 0.102), diabetes (100% vs. 100%, P = 0.418), and elderly age (100% vs. 100%, P = 0.149).

## Discussion

This study investigated the efficacy and safety of atezolizumab plus bevacizumab therapy for uHCC in real-world practice. The ORR and DCR were 22.0% and 70.6%, respectively, and the median PFS was 5.7 months in the whole cohort. Clinical factors associated with worse PFS were younger age, a higher number of intrahepatic tumors, macrovascular invasion, and higher NLR. This study revealed the therapeutic outcomes and predictive factors for atezolizumab plus bevacizumab therapy in the prospective cohort registered before treatment.

Previous small-group retrospective studies on atezolizumab plus bevacizumab therapy reported an ORR and DCR for the first systemic treatment of 24.0–50.0% and 57.7–66.6%, respectively [6, 7, 9, 11, 23–27]. In addition, Japanese studies reported a median PFS of approximately 8.0–9.0 months for the first systemic treatment [27, 28]. The ORR, DCR, and PFS in these previous retrospective studies were similar to those of the IMbrave 150 trial, which reported an ORR and DCR based on RECIST version 1.1 of 27.3% and 73.6%, respectively, and a median PFS of 6.9 months [1]. The present study revealed that the therapeutic results in real-world practice. The efficacy was comparable to the results of the IMbrave 150 trial although the present study included patients who received atezolizumab plus bevacizumab therapy not only as first systemic treatment but also second-line or later systemic treatment.

The present prospective study revealed that age ≥75 years was associated with prolonged PFS in patients treated with atezolizumab plus bevacizumab therapy. In contrast, previous studies with shorter observation periods investigating the tolerability and efficacy of atezolizumab plus bevacizumab therapy in elderly patients observed no significant difference in PFS between younger and older patients [2, 29]. However, other previous studies reported better rates of response to ICIs in older patients with melanoma and showed that younger patients had a higher expression of regulatory T cells than older patients [30, 31]. Furthermore, CD8+ effector T cells were reduced in younger patients. This is responsible for the difference in the therapeutic responses between younger and older patients [31]. Such immune change in the tumor microenvironment also reduces the therapeutic benefit of atezolizumab plus bevacizumab therapy for HCC [32]. Additionally, age-related changes in the tumor microenvironment may affect the therapeutic response of HCC. To our knowledge, the present study is the first to report on the association between older age and better therapeutic efficacy of ICIs for uHCC. The results of this prospective study suggest that atezolizumab plus bevacizumab therapy may be preferred when selecting the systemic treatment regimen for elderly patients with uHCC. In contrast, this study observed no difference in OS between the older and younger patients. Ando et al. reported that subsequent systemic treatment was associated with the prognosis after first-line systemic treatment in patients with HCC [21]. After discontinuing atezolizumab plus bevacizumab therapy for HCC, some multikinase inhibitors, such as sorafenib and lenvatinib, could be used as subsequent treatment. Regarding the association between intolerance for multikinase inhibitors and age, Kinoshita et al. reported that a lower dose intensity causing poor therapeutic efficacy was observed in patients aged ≥80 years treated with lenvatinib for HCC [33]. The intolerance to subsequent multikinase inhibitors might contribute to the unimproved prognosis in elderly patients in this study. However, the median OS was not reached, and further long-term observation on OS is required.

Of the other clinical indicators associated with therapeutic efficacy in this study, NLR was a valid inflammatory parameter induced by tumor growth or microenvironment [34]. Recently, NLR has been identified as a factor that can predict therapeutic outcomes for various solid tumors, including HCC [35–37]. In addition, some studies reported that NLR was a prognostic factor in HCC treated with ICIs, such as atezolizumab plus bevacizumab [7, 19, 23, 38] and nivolumab [39]. The present prospective study confirmed that NLR was certainly associated with PFS.

This study has some limitations. First, the observation period was insufficient to analyze OS. Only approximately a quarter of the study patients died, and the median OS had not been reached. Second, only indicators used in routine clinical practice were assessed in this study without considering factors such as biological markers and genetic predisposition.

In conclusion, this multicenter prospective observational study suggested that younger age under 75 years, more than four intrahepatic tumors, macrovascular invasion, and elevated NLR of more than 3.03 would be risk factors for the shortened PFS in atezolizumab plus bevacizumab therapy for uHCC.

## Supporting information

S1 Fig Therapeutic efficacy of atezolizumab plus bevacizumab therapy (a) and PFS (b) and OS (c) of patients in the first-line systemic treatment group.
(TIF)

S2 Fig Therapeutic efficacy (a) and PFS (b) based on mRECIST in all patients. Therapeutic efficacy (c) and PFS (d) based on mRECIST in the first-line systemic treatment group.
(TIF)

S1 Table The frequency of severe AEs. (DOCX)

S1 Data (XLSX)
