# Supplementary figures and images for: Clinical factors associated with the therapeutic efficacy of atezolizumab plus bevacizumab in patients with unresectable hepatocellular carcinoma: A multicenter prospective observational study
Source: PLoS One. 2024 Jan 2;19(1):e0294590. doi: 10.1371/journal.pone.0294590 (PMC10760712; doi:10.1371/journal.pone.0294590)

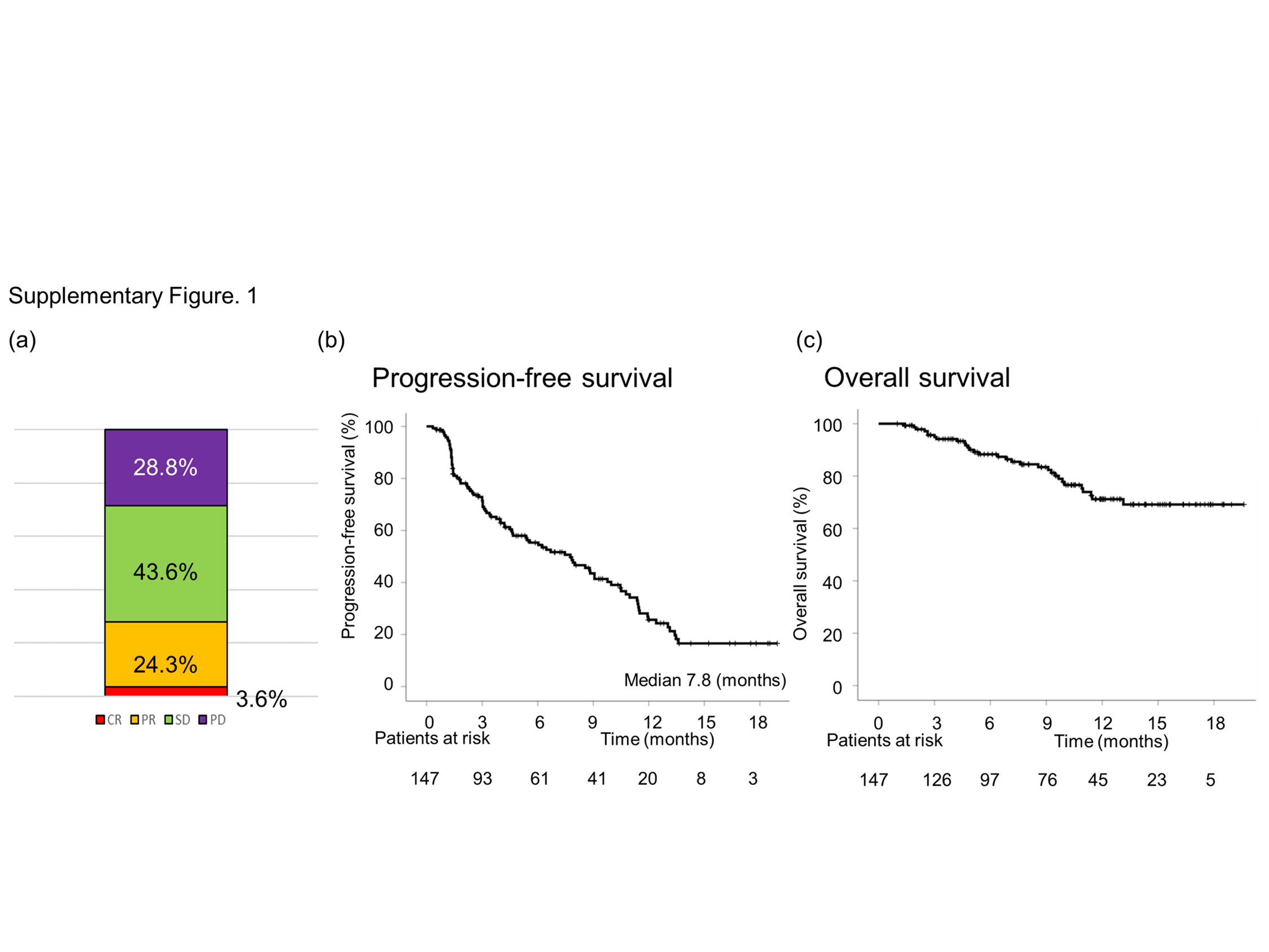

Supplement: S1 Fig — Therapeutic efficacy of atezolizumab plus bevacizumab therapy (a) and PFS (b) and OS (c) of patients in the first-line systemic treatment group. (TIF) [file pone.0294590.s001.tif]

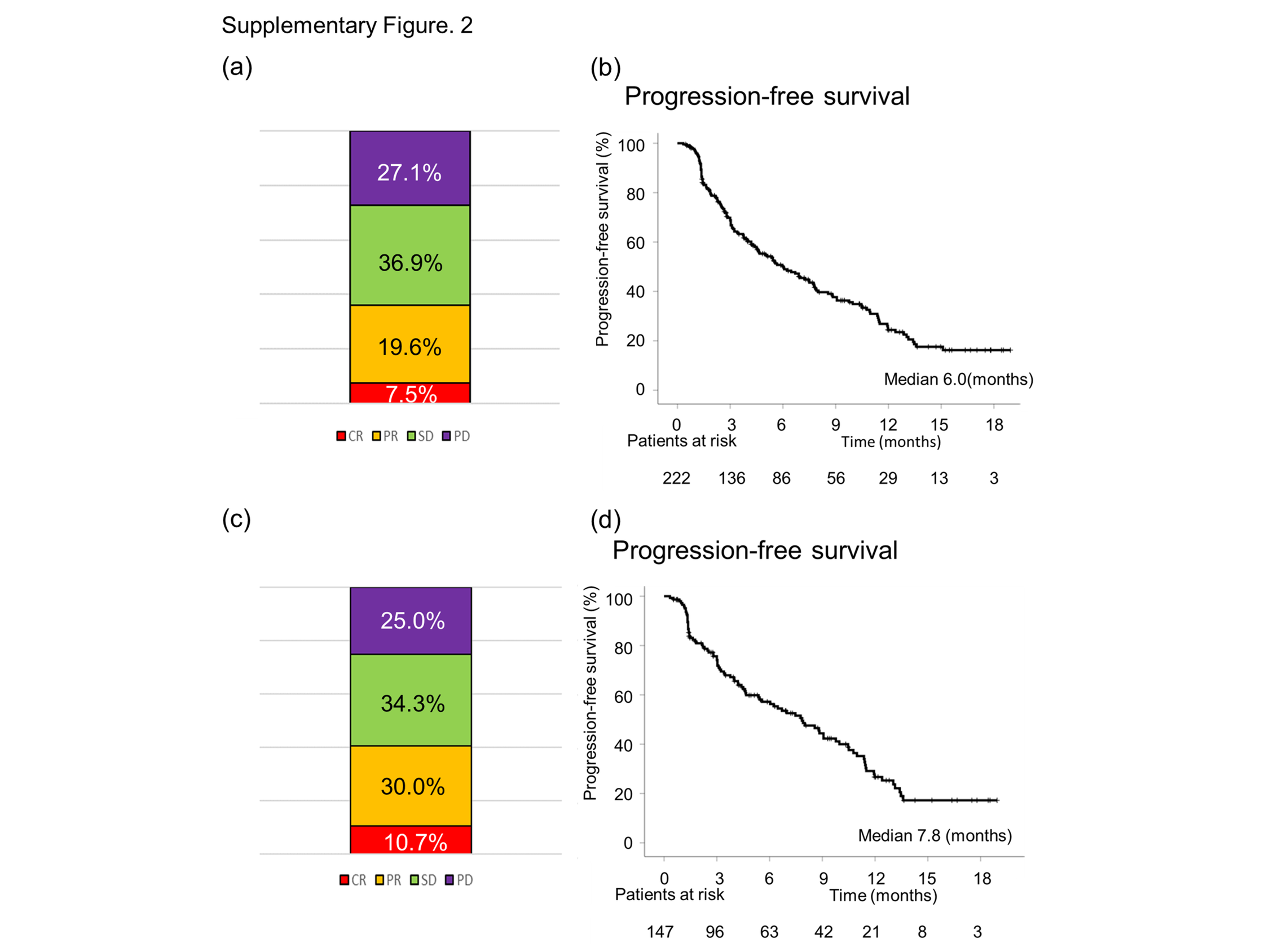

Supplement: S2 Fig — Therapeutic efficacy (a) and PFS (b) based on mRECIST in all patients. Therapeutic efficacy (c) and PFS (d) based on mRECIST in the first-line systemic treatment group. (TIF) [file pone.0294590.s002.tif]
