# Supplementary material for: Clinical factors associated with the therapeutic efficacy of atezolizumab plus bevacizumab in patients with unresectable hepatocellular carcinoma: A multicenter prospective observational study
Source: PLoS One. 2024 Jan 2;19(1):e0294590. doi: 10.1371/journal.pone.0294590 (PMC10760712; doi:10.1371/journal.pone.0294590)
Supplement: S1 Table — (DOCX) [file pone.0294590.s003.docx]

**Supporting Table1.** The frequency of severe AEs

|  | **No severe AEs**  **n = 142** | **Severe AEs**  **n = 80** | **P value** |
| --- | --- | --- | --- |
| Age  <75, n (%)  ≥75, n (%) | 80 (65.6)  62 (62.0) | 42 (34.4)  38 (38.0) | 0.674 |
| Hypertension  Absent, n (%)  Present, n (%) | 41 (61.2)  101 (65.2) | 26 (38.8)  54 (34.8) | 0.648 |
| Diabetes Mellitus  Absent, n (%)  Present, n (%) | 90 (66.7)  52 (59.8) | 45 (33.3)  35 (40.2) | 0.318 |
| Hyperlipidemia  Absent, n (%)  Present, n (%) | 112 (61.2)  30 (75.0) | 70 (38.8)  10 (25.0) | 0.145 |
| Prior systemic therapy  Absent, n (%)  Present, n (%) | 101 (68.7)  41 (54.7) | 46 (31.3)  34 (45.3) | 0.054 |
